# EMT Molecular Signatures of Pancreatic Neuroendocrine Neoplasms

**DOI:** 10.3390/ijms232113645

**Published:** 2022-11-07

**Authors:** Abhirami Venugopal, Agnes Michalczyk, Mustafa Khasraw, M. Leigh Ackland

**Affiliations:** 1 Centre for Cellular and Molecular Biology, School of Life and Environmental Sciences, Deakin University, 221 Burwood Highway, Burwood, VIC 3125, Australia; 2 Duke University Medical Center, Durham, NC 27710, USA

**Keywords:** neuroendocrine neoplasms (NEN), biomarkers, qRT-PCR, immunohistochemistry, epithelial to mesenchymal transition (EMT)

## Abstract

Neuroendocrine neoplasms (NENs) are relatively rare neoplasms occurring predominantly in the gastrointestinal tract and pancreas. Their heterogeneity poses challenges for diagnosis and treatment. There is a paucity of markers for characterisation of NEN tumours. For routine diagnosis, immunohistochemistry of the NEN-specific markers CgA and synaptophysin and the proliferation marker Ki-67 are used. These parameters, however, are qualitative and lack the capacity to fully define the tumour phenotype. Molecules of epithelial–mesenchymal transition (EMT) are potential candidates for improved tumour characterisation. Using qRT-PCR, we measured mRNA levels of 27 tumour markers, including 25 EMT-associated markers, in tumour tissue and matched non-tumour tissues for 13 patients with pancreatic NENs. Tissue from patients with three different grades of tumour had distinctly different mRNA profiles. Of the 25 EMT-associated markers analysed, 17 were higher in G3 tissue relative to matched non-tumour tissue, including CD14, CD24, CD31, CD44, CD45, CD56, CK6, CK7, CK13, CK20, NSE, CDX2, CgA, DAXX, PCNA, laminin and Ki-67. The differences in levels of seven EMT-associated markers, Ki-67, DAXX, CD24, CD44, vimentin, laminin and PDX1 plus CgA and NSE (neuroendocrine markers) enabled a distinct molecular signature for each tumour grade to be generated. EMT molecules differentially expressed in three tumour grades have potential for use in tumour stratification and prognostication and as therapeutic targets for treatment of neuroendocrine cancers, following validation with additional samples.

## 1. Introduction

Neuroendocrine neoplasms (NENs) are a relatively rare, heterogeneous family of tumours that occur mainly in the gastrointestinal tract (62–67%) and lung (22–27%), with 7% located in the pancreas [1,2]. Tumours are termed functional if they are associated with secretion of specific hormones, including insulin, glucagon and VIP hormone, with some causing symptoms such as facial flushing, diarrhoea, while nonfunctional tumours may show no overt signs of hormone-associated disease secretion [3]. Functional tumours may be identified early as they are accompanied by changes in hormonal levels; however, 90% of neuroendocrine tumours are nonfunctional and are frequently not diagnosed until a late stage where patients may present with nonspecific symptoms, including abdominal pain, palpable mass, fatigue, etc. [4] or by chance on cross-sectional imaging for other conditions. Factors contributing to tumour heterogeneity can pose many clinical challenges for diagnosis and treatment [5,6].

Routine diagnosis of NENs is based on tissue biopsy of chromogranin A (CgA), a protein found exclusively in the secretary granules of endocrine and neuroendocrine cells [7,8], and synaptophysin, an integral membrane protein of neurotransmitter vesicles present in primary and metastatic neuroendocrine tumour cells [9].

NENs are graded based on the mitotic index using Ki-67, a marker for proliferation, and using immunohistochemistry (IHC) as a diagnostic tool. Ki-67 is a nuclear antigen present at all stages of the cell cycle except the G0 phase [10]. It was used to assess the proliferative status in lung and breast cancer in the late 1980s [11] and is considered to be a reliable marker that can be reproducibly used across laboratories [12]. Based on the 2019 WHO classification, NENs are classified into three grades: Grade 1 (low grade) with a mitotic rate <2 mitoses/2 mm^2^ and Ki-67 < 3% (NET, G1), Grade 2 (intermediate) with a mitotic rate of 2–20 mitoses/2 mm^2^ and Ki-67 of 3–20% (NET, G2) and Grade 3 (high) with mitotic rates of >20 mitoses/2 mm^2^ and Ki-67 > 20% (NET, G3) [13]. Neuroendocrine carcinomas (NEC’s) are small cell type or large cell type, both poorly differentiated and with mitotic rate and Ki-67 > 20%.

Although Ki67, CgA and synaptophysin have been used for regular diagnosis of NETs, there have been studies questioning the reliability of these markers and the basis of their use for treatment decisions. Inter- and intra-observer variability in the counting of Ki-67 has been noted, with a caution to pathologists to be aware while grading [14]. A major problem with the existing markers is that as single analytes, most lack the capacity to define the various characteristics associated with tumour cells and the origin of the primary site in metastatic disease. The liver is often a common site of secondary lesions in neuroendocrine cancer, thus, knowing the primary origin may assist in prognosis and tumour management [15]. Hence, there is a requirement for markers to further characterise tumour origin, grade and prognosis [6].

To date, pathological reports of NETs have been based on the use of a small number of markers using IHC and only on tumour tissue without taking into account matched non-tumour tissue. The paucity of markers in NET management has remained a significant problem. The New Multinational and Multidisciplinary Delphi Consensus meeting of a team of NEN experts concluded the need for novel makers as one of the key gaps that needs to be addressed [16]. This calls for the development of marker panels that have the capacity to distinguish the various types of NETs and that comply with the definition of a biomarker in being highly sensitive and specific to tumour types. IHC is the most widely used diagnostic procedure but it is a qualitative technique and is prone to variability between labs which predominantly comes from antigen retrieval and staining procedures, as seen with a multicentric study for the Ki-67 labelling index [17].

The epithelial–mesenchymal transition (EMT) is a collection of cellular changes that are a critical during development. Inappropriate activation of the EMT in cancer accounts for tumour dissemination from the primary to secondary sites [18]. It provides a basis for understanding cancer metastasis. During EMT, epithelial cells lose their cell–cell adhesion properties and gain migratory and invasive characteristics to become mesenchymal cells [19,20]. Numerous signalling pathways control EMT, including some that overlap with cell growth, and stem cell proliferation and differentiation/de-differentiation [21]. EMT occurs as a gradual process, characterised by intermediate cellular states that express different levels of epithelial and mesenchymal markers and exhibit intermediate morphological features, between epithelial and mesenchymal cells [22]. While the molecular pathways underlying EMT have been well-characterised in breast cancer and breast cancer cell culture models [23], less is known about the EMT processes in neuroendocrine tumours.

In a previous study, to improve the definition of tumour phenotypes, we tested a panel of 23 tumour-associated markers to profile matched tumour tissue relative to non-tumour tissue from a patient with a G1 pancreatic neuroendocrine tumour [24]. We utilised qRT-PCR, a quantitative diagnostic tool for differential marker profiling, that has the potential, and when used in combination with IHC has potential clinical utility, towards improving tumour stratification. We identified a panel of 11 markers associated with EMT that indicated that cells within the NEC tissue had transitioned to a mesenchymal phenotype.

In the current study, we measured mRNA levels of the panel of 11 EMT-associated markers in tissue from patients with grade 1, 2 and 3 pancreatic neuroendocrine tumours (pNETs) and generated a molecular profile specific to each tumour grade.

## 2. Results

### 2.1. mRNA Levels of Tumour-Associated Markers within Each Grade

mRNA levels of 27 tumour-associated markers were measured in tumour tissue relative to non-tumour in all 13 patients grouped into Grade 1, Grade 2 and Grade 3 (Table 1). Relative to the non-tumour tissue, the mRNA marker levels of the majority of individual samples showed similar trends within each of the three grades. In G1 patients, CgA, CD56, PDX1 and p53 were highly expressed, except for CgA and CD56 in one patient. CD24, CD44, laminin, CD49, CDX2, CK6 and DAXX had lower expression in the tumour relative to that in the non-tumour tissue (Table 1). In G2 patients, with the exception of Ki-67 in one patient, CgA, CD56, Ki-67, CD31 and vimentin were more highly expressed in tumour tissue relative to that in non-tumour tissue. Markers CD24, CD44, PDX1, synaptophysin, CD49, CDX2, CD45, DAXX, PCAD and CK7 had lower expression in the tumour relative to that in the non-tumour with the exception of one patient (Table 1). In G3 patients, CgA, CD56, Ki-67, CD24, CD44 and laminin were highly expressed in tumour tissue relative to that in non-tumour tissue with the exception of CD24 for one patient. Markers vimentin, synaptophysin, MENA, ECAD and CK20 had lower expression in the tumour tissue relative to that in the non-tumour tissue with the exception of MENA for one patient and CK20 for another patient (Table 1).

The outlier samples that did not fit in the overall pattern seen within each grade included CgA and CD56 in Grade 1 which were lower in Patient 1 relative to that in non-tumour tissue, while the other G1 tissues were higher in the tumour tissues relative to those in the non-tumour tissue. Within G2, Ki-67 was lower in Patient 2, and CD24, CDX2 and CD45 were higher in Patient 1 relative to those in the non-tumour tissue, in contrast to the other tissues. In Patients 1 and 3, CD44 and CD49 were high in two samples and lower in the remaining three tissues, relative to those in the non-tumour tissue. In G3 samples, CD24 and laminin were lower in Patient 4 and in contrast to the other G3 samples, while vimentin was higher in Patient 3, ECAD higher in Patient 2 and CK20 higher in Patient 4 relative to those in the non-tumour tissue and in contrast to the other G3 samples.

### 2.2. Mean Marker Profile between Tumour and Non-Tumour Tissue within NEN Grades

**Figure 1:**
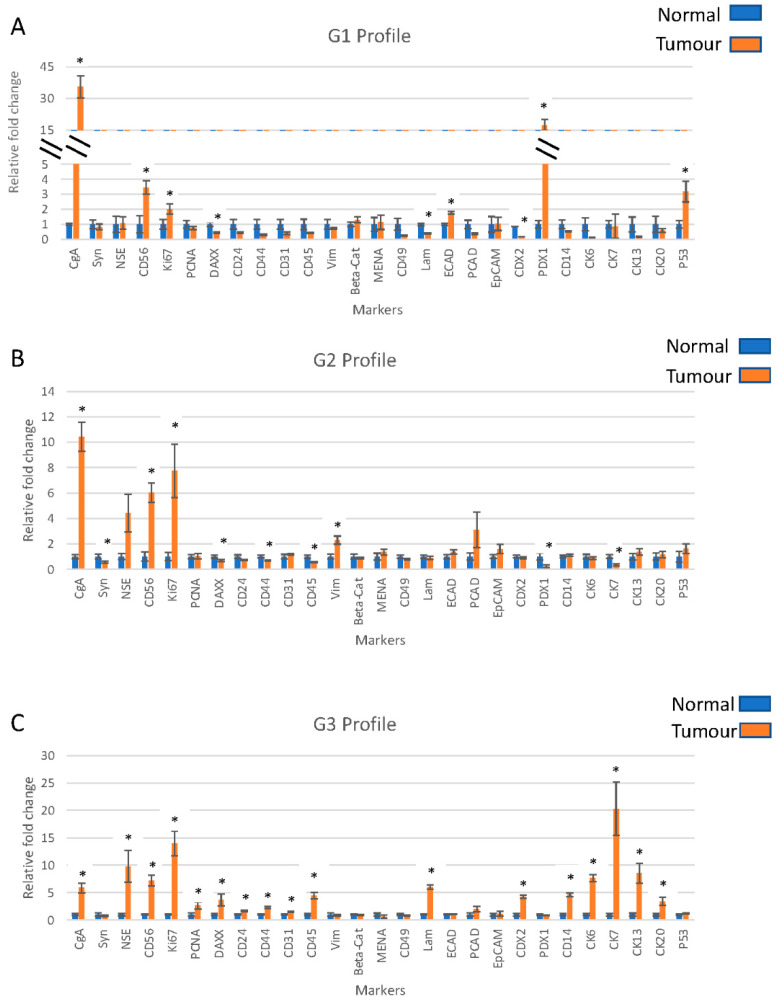
mRNA marker profiles for each of grade 1 (**A**), grade 2 (**B**) and grade 3 (**C**) pancreatic neuroendocrine tumours. These profiles were developed using the average expression of all the samples from each grade. All marker expressions were normalised to the matched non-tumour tissues and using β-actin as the endogenous control. * represents a statistically significant difference (*p* < 0.05) in the marker expression in the tumour relative to that in non-tumour.

As the majority of markers within each grade showed consistent trends, the average mRNA marker levels were determined. In G1, the mRNA levels of CD56, CgA, ECAD, P53, PDX1 and Ki-67 were higher in tumour tissue relative to those in non-tumour tissue and CDX2, DAXX and laminin showed lower expression levels in tumour tissue (Figure 1A). In G2, CD56, CgA, NSE, vimentin and Ki-67 were higher in tumour tissue compared to those in non-tumour, and levels of CD44, CD45, CK7, DAXX, synaptophysin and PDX1 were lower in tumour tissue relative to those in non-tumour tissue (Figure 1B). In G3, CD14, CD24, CD31, CD44, CD45, CD56, CK6, CK7, CK13, CK20, NSE, CDX2, CgA, DAXX, PCNA, Laminin and Ki-67 were higher in tumour tissue relative to those in non-tumour (Figure 1C). Markers with >5-fold difference in G3 tumour relative to non-tumour tissue included CD56 (7.2-fold increase), CK6 (7.7-fold increase), CK7 (20.3-fold increase), CK13 (8.5-fold increase), NSE (9.8-fold increase), CgA (5.9-fold increase), laminin (6-fold increase) and Ki-67 (14-fold increase). All markers that showed increased expression in G3 tumour tissue, with the exception of CgA, also had higher levels in G1 and G2 tumour tissues relative to those in the respective non-tumours.

### 2.3. Comparison of Tumour Tissue Marker Levels across Grades

**Figure 2:**
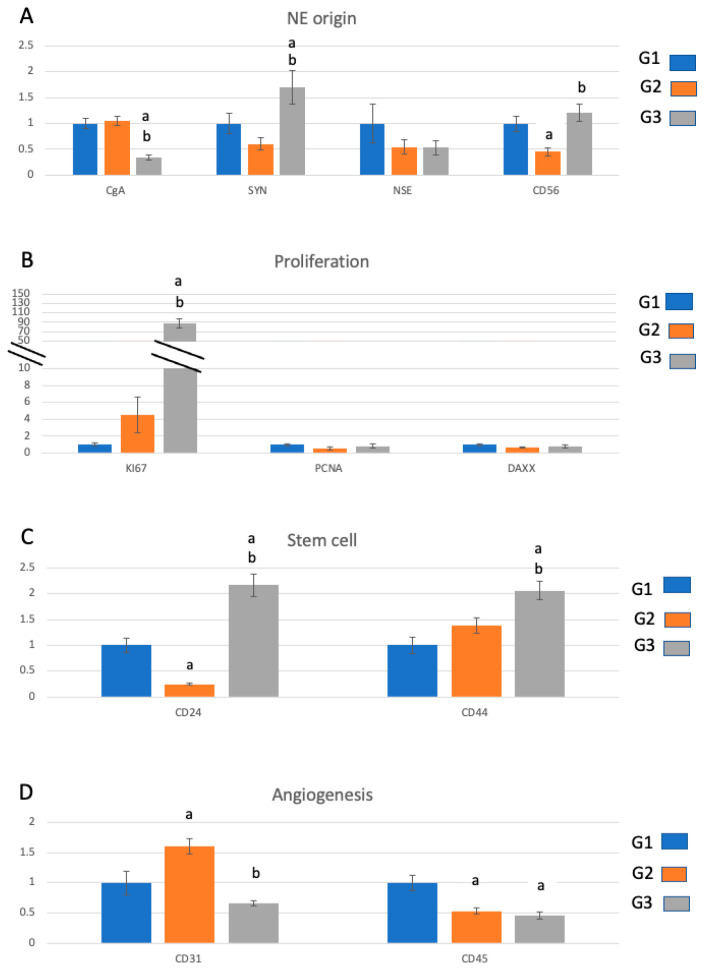

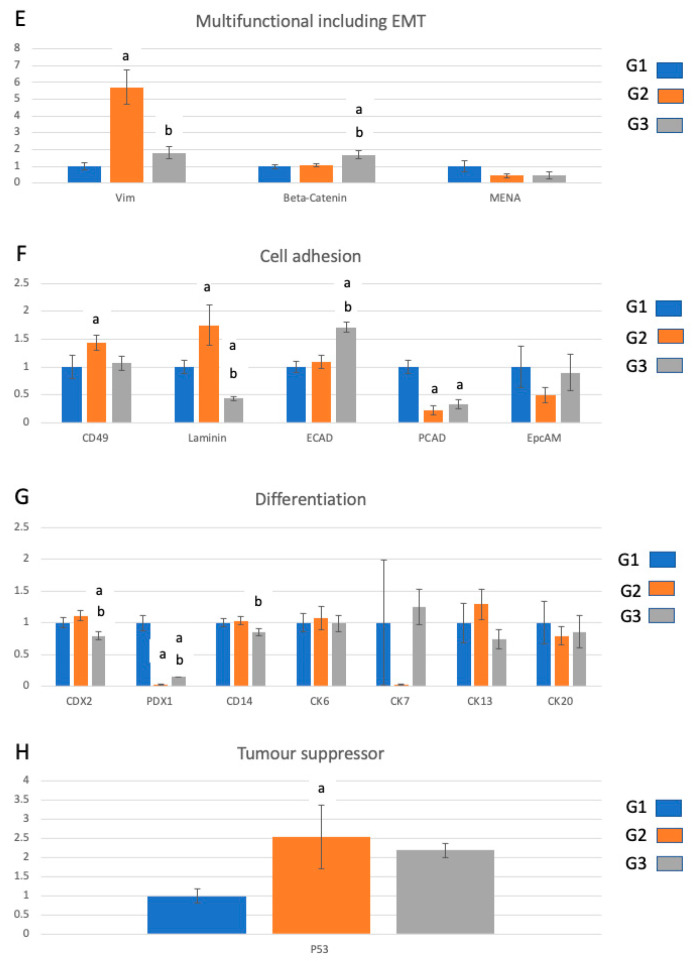
mRNA marker profiles in the tumour tissue across grades. The markers have been grouped according to the tumour characteristic: NE origin (**A**), proliferation (**B**), stem cell (**C**), angiogenesis (**D**), multifunctional including EMT (**E**), cell adhesion (**F**), differentiation (**G**), tumour suppressor (**H**). The marker expressions in G2 and G3 have been normalised to the average of the G1 samples. “a” represents a statistically significant difference (*p* < 0.05) in marker expression in the tumour relative to that in the G1 tumour. “b” represents a statistically significant difference (*p* < 0.05) in marker expression in the G3 tumour relative to that in the G2 tumour.

As most reports present tumour data without matching non-tumour controls, we compared mRNA marker levels in tumour tissue only, between different grades (Figure 2). The mRNA levels in G2 and G3 were normalised to G1 and grouped based on tumour characteristics. Within the markers for neuroendocrine origin, CgA was lower in G3 relative to that in G1 and G2, and synaptophysin was higher in G3 relative to that in G1 and G2, while CD56 displayed higher levels in G3 and lower levels in G2 relative to those in G1 (Figure 2A). NSE showed no differences between grades. Within the markers of proliferation, Ki-67 showed the highest levels in G3 relative to that in G1 and G2, while PCNA and DAXX showed no differences between grades (Figure 2B). Stem cell markers CD24 and CD44 were higher in G3 than in G1, although CD24 was lower in G2 than in G1 (Figure 2C). The marker of angiogenesis CD31 was the highest in G2 and the lowest in G3, while CD45 had lower levels in both G2 and G3 relative to that in G1 (Figure 2D).

Among the EMT markers, vimentin was most highly expressed in G2 relative to that in G1 tissue (Figure 2E). b-catenin was the highest in G3, while G1 and G2 showed similar expression levels. There were no differences between grades for MENA (Figure 2E). Among the cell adhesion markers, CD49 was high in G2, but there was no difference between G1 and G3. Laminin was most highly expressed in G2 and lowest in G3 relative to that in G1. ECAD was the highest in G3, but no differences between G1 and G2 were detected (Figure 2F). PCAD was lower in G2 and G3 than in G1, and no differences in EpCAM expression were seen between grades (Figure 2F).

The markers of differentiation CDX2 and CD14 were the lowest in G3 with no differences between G1 and G2. PDX1 was lower in both G2 and G3 relative to that in G1, and for CK6, CK7, CK13 and CK20, no differences were seen between grades (Figure 2G).

The tumour suppressor marker p53 was the highest in G2, and no differences between G1 and G3 were observed (Figure 2H).

### 2.4. Immunohistochemical Expression Pattern of Markers

**Figure 3:**
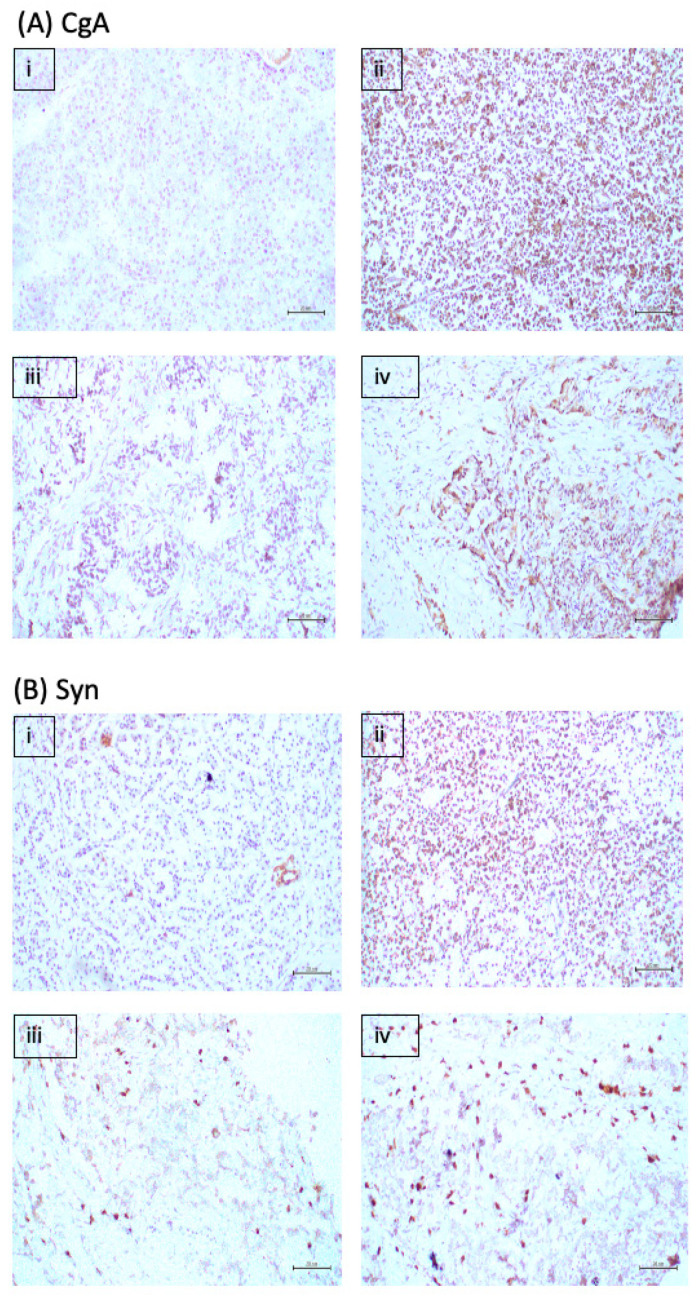

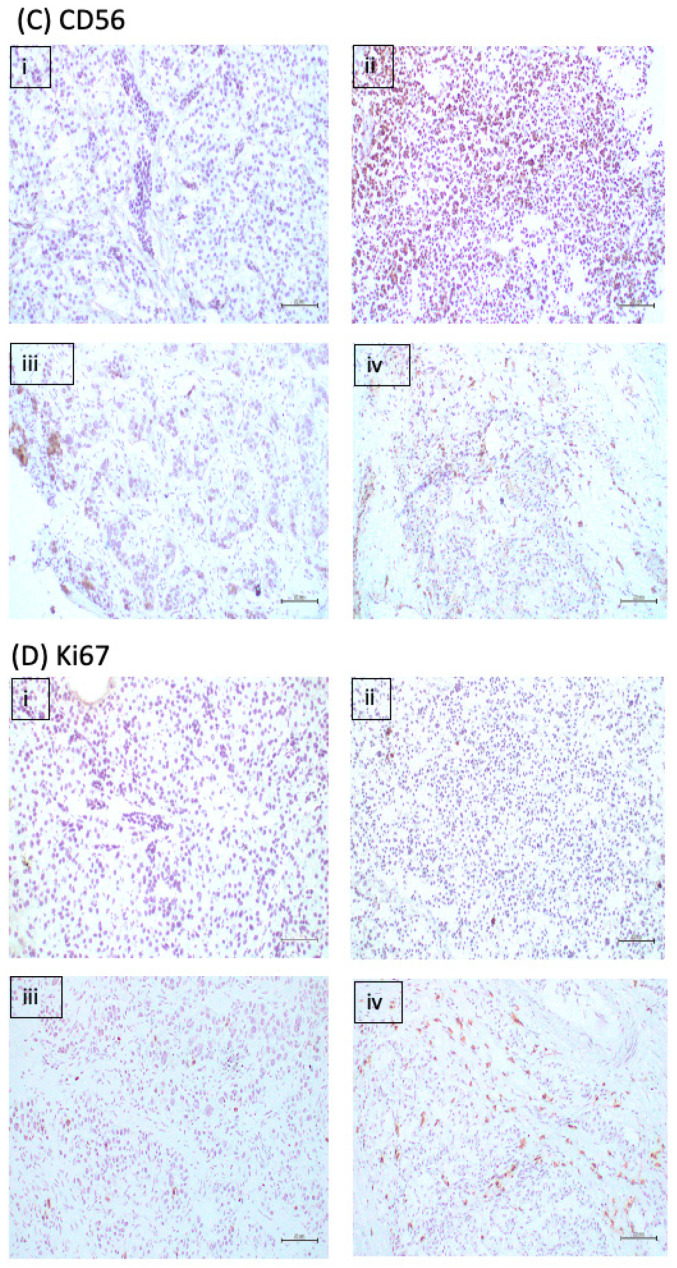

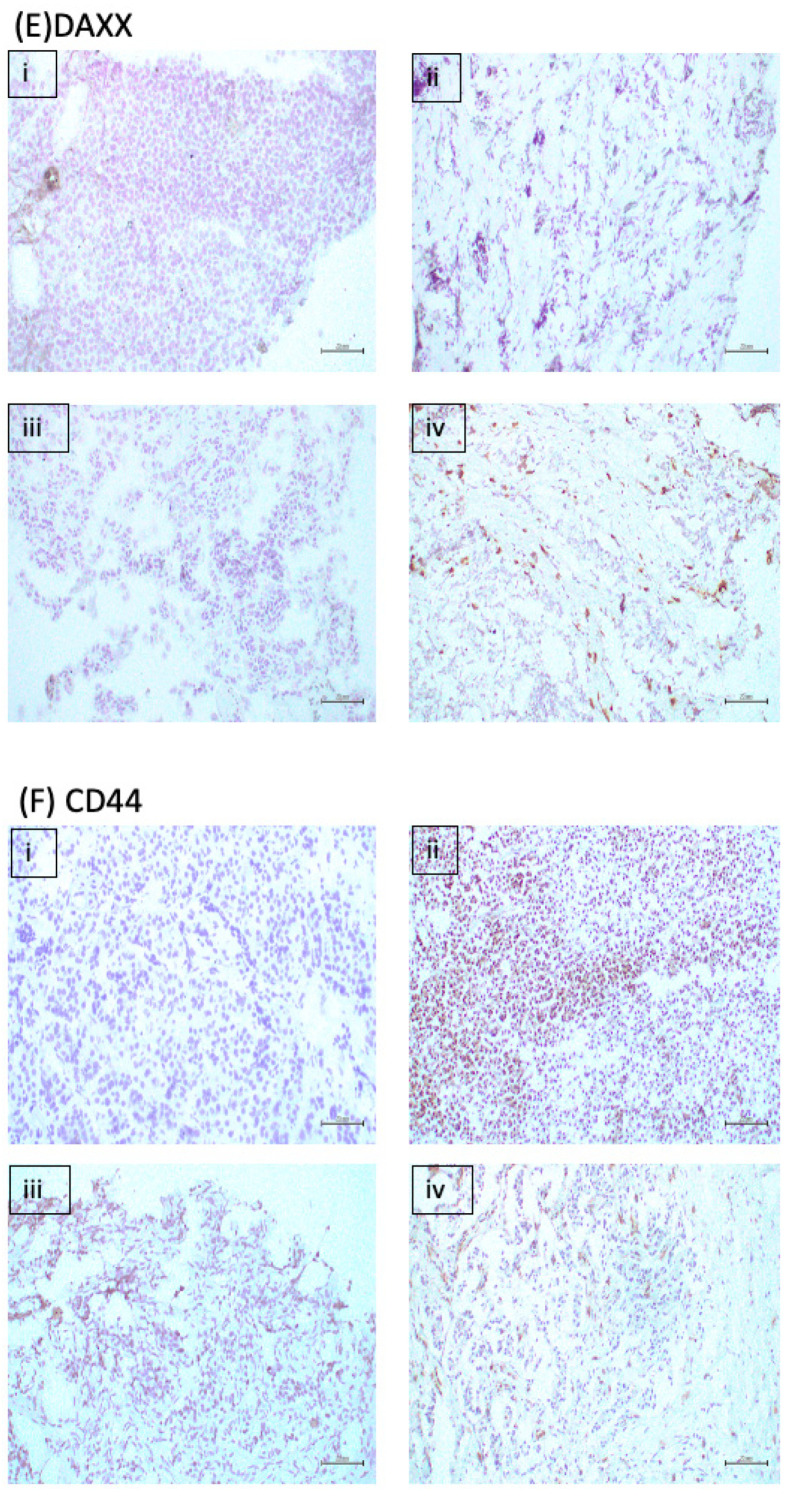

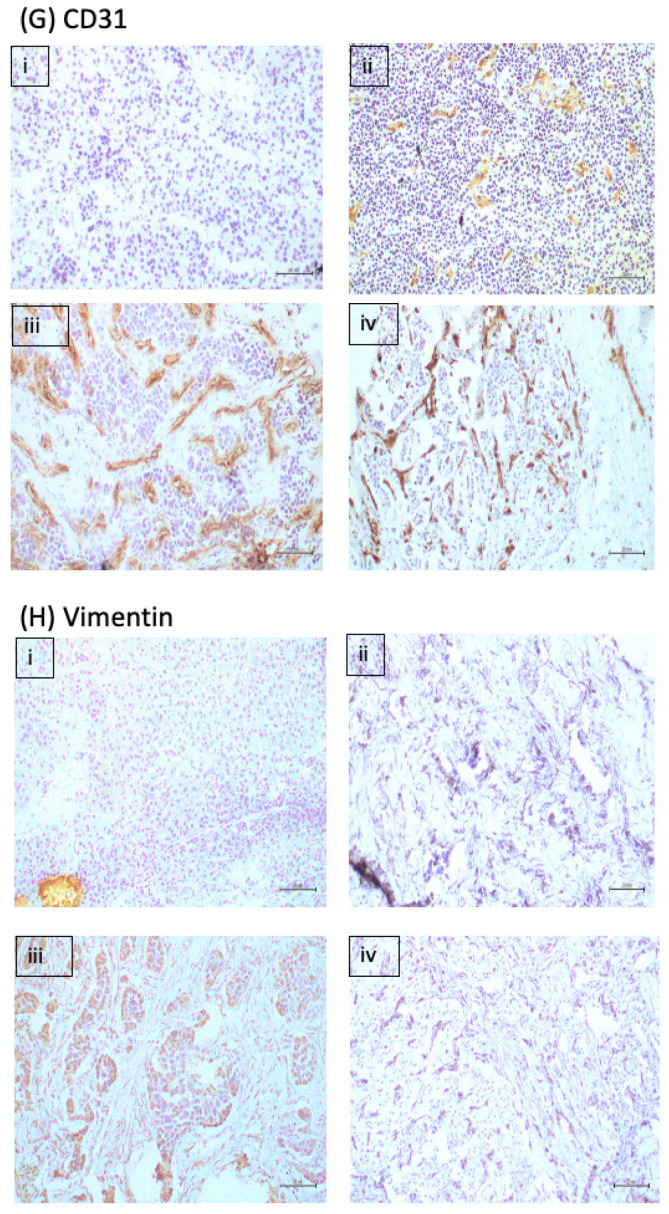

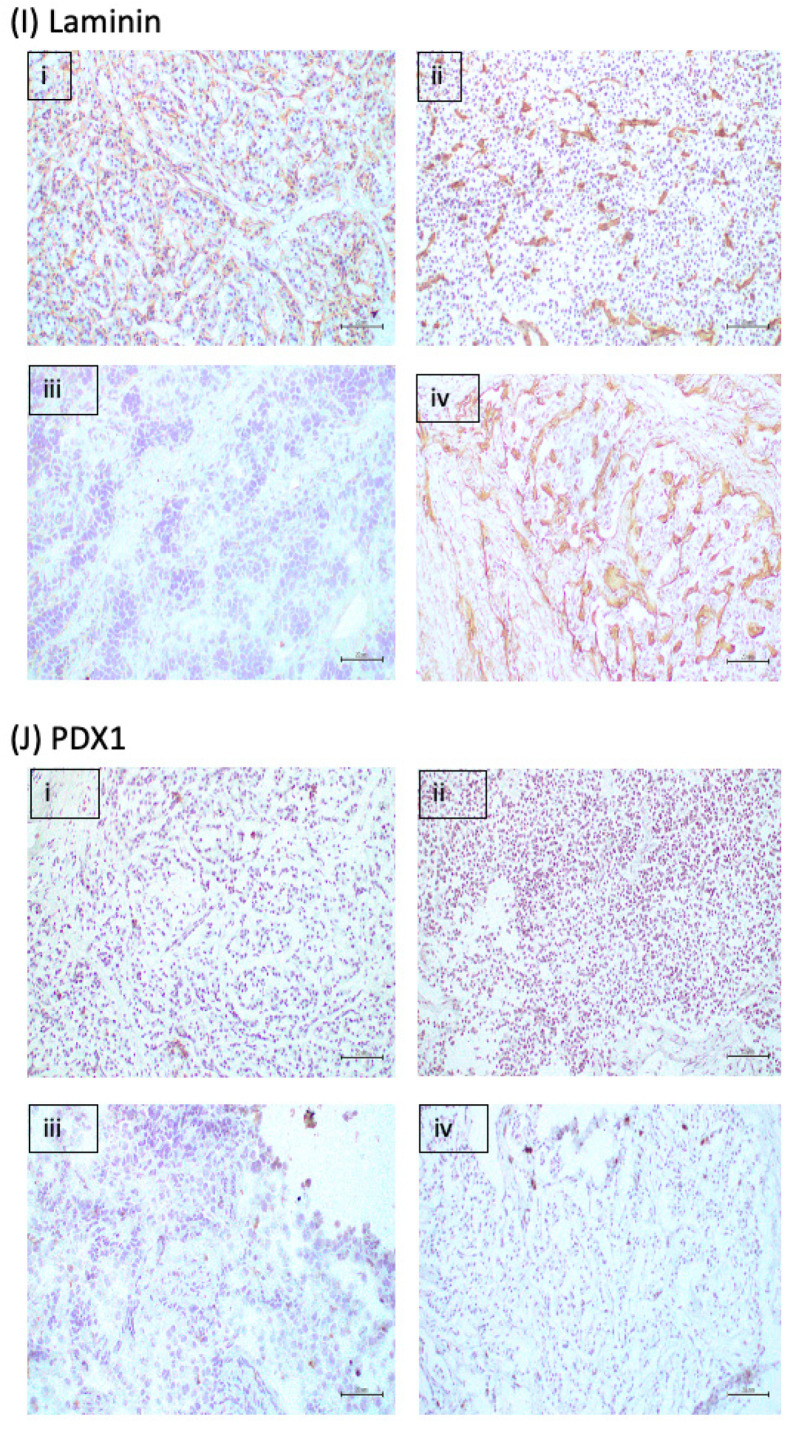

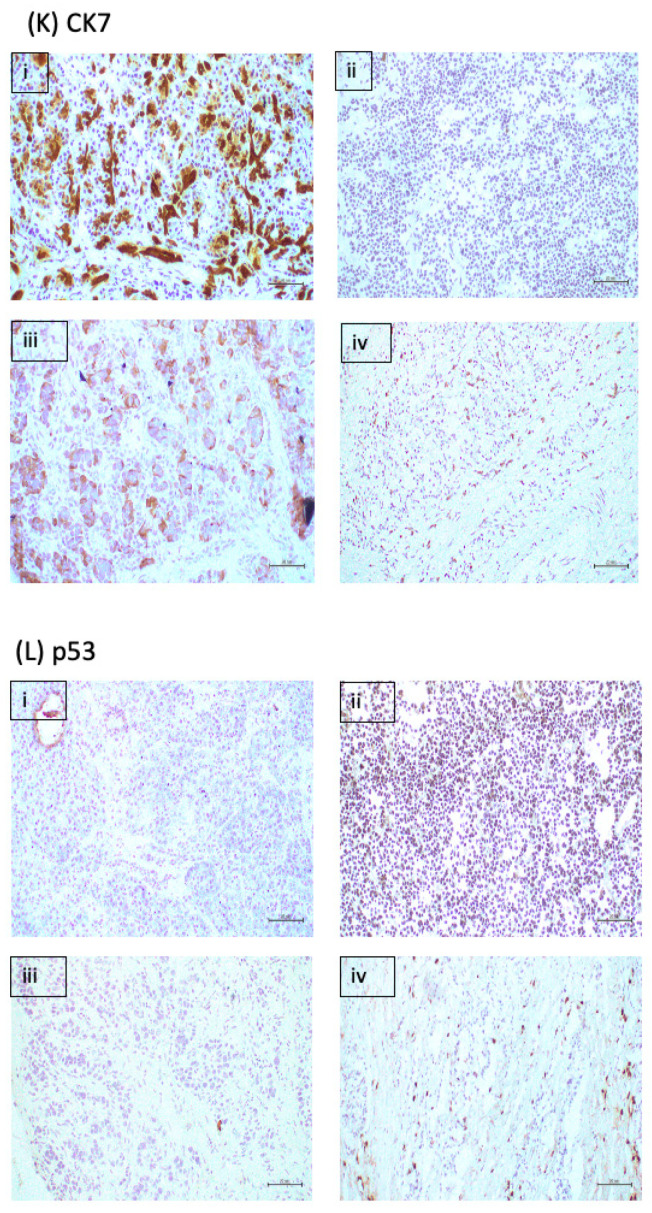
IHC images for selected markers (**A**) CgA, (**B**) synaptophysin, (**C**) CD56, (**D**) Ki-67, (**E**) DAXX, (**F**) CD44, (**G**) CD31, (**H**) vimentin, (**I**) laminin, (**J**) PDX1, (**K**) CK7, (**L**) p53 at 200× magnification. For each marker, the four images represent (i) non-tumour pancreatic tissue, (ii) G1, (iii) G2 and (iv) G3 tumour tissue. Scale: 20 μm.

Immunohistochemical analysis indicated that CgA expression was absent in non-tumour tissue, while stained cells represented 25–50% in G1, less than 25% in G2 and 25–50% in G3 (Figure 3A(i–iv)). Synaptophysin staining was absent in the non-tumour tissue, and in G1, stained cells represented 25–50% of cells, while in G2 and G3, intense staining was found in less than 25% of tumour cells (Figure 3B(i–iv)). CD56 was absent in non-tumour tissue, but strong staining was present in 50% of G1 tumour cells and in less than 25% G2 and G3 cells (Figure 3C(i–iv)). Ki-67 staining was absent in non-tumour tissue and was progressively higher in G1, G2 and G3 tumour tissue where it showed nuclear localisation (Figure 3D(i–iv)).

DAXX staining was absent in non-tumour, G1 and G2 tumour tissues, while strong nuclear staining was observed in less than 25% of cells in G3 tumour tissue (Figure 3E(i–iv)). CD44 staining was absent in the non-tumour tissue; however, strong staining was observed in 25–50% of cells in G1 tissue, with weak staining was visible in G2 and strong, focal staining in a small proportion of cells in G3 tissue (Figure 3F(i–iv)). CD31 was absent in non-tumour tissue but present in G1, G2 and G3 tumour tissues (Figure 3G(i–iv)). Vimentin was absent in non-tumour tissue with weak staining seen in G1 and G3 tissue, while 25–50% of G2 cells were stained (Figure 3H(i–iv)).

In non-tumour tissue 25–50% of cells were strongly stained with laminin with less staining in G1 tumour cells, while staining was absent in G2 but strong in more than 50% of G3 cells (Figure 3I(i–iv)). PDX1 was observed in few cells in non-tumour tissue, while 50% of cells were stained in G1 and few cells stained in G2 and G3 tissue (Figure 3J(i–iv)). Intense CK7 staining was seen in over 50% of cells in the non-tumour tissue, no staining was detected in G1, but staining was observed in 25% of cells in G2 and G3 tissues (Figure 3K(i–iv)). P53 was absent in non-tumour tissue, and intense staining was observed in 25–50% cells in G1, while weak staining was found in a few G2 cells, and in less than 25% of G3 cells (Figure 3L(i–iv)).

### 2.5. Comparison of mRNA and Protein Levels for the Selected Markers

A comparison was made between the mRNA levels and the IHC and protein labelling for the 12 markers that showed the greatest differences in mRNA levels between tumour and non-tumour tissue (Table 2). Correlation between mRNA and protein labelling was found for CgA, CD56, Ki-67 and laminin in all three grades. mRNA and protein levels correlated for DAXX (G3), CD44 (G3) and CD31 (G2, G3), vimentin (G2), laminin, PDX1 (G1, G2), CK7 (G1, G2) and p53 (G1, G3). mRNA and protein levels of the markers synaptophysin, DAXX (G1, G2), CD44 (G1, G2), CD31 (G1), vimentin (G1, G3), PDX1 (G3), CK7 (G3) and p53 (G2) did not correlate.

### 2.6. Molecular Signature

**Figure 4:**
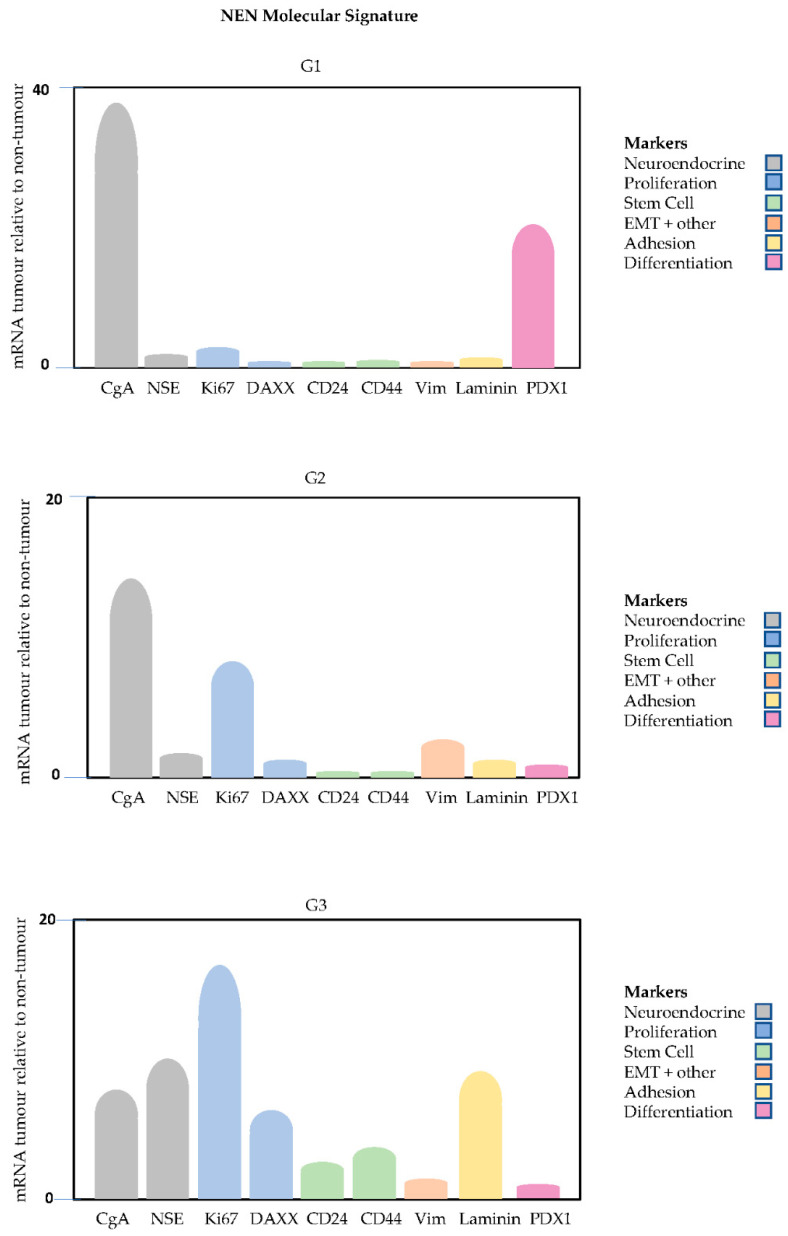
Molecular fingerprint depicting the pattern of the mean mRNA levels in tumour tissue relative to those in non-tumour for each marker across three grades for different categories of markers including neuroendocrine, proliferation, stem cell, multifunctional including EMT (EMT+), adhesion and differentiation.

The differences in marker profiles between the three tumour grades relative to non-tumour tissue can be illustrated diagrammatically, where the differences in the mean mRNA levels for each marker across three grades are shown (Figure 4). This enables the different marker profiles between the grades to be clearly distinguished and shows the marker trends.

## 3. Discussion

Classification and grading for NEN subtypes will benefit from more reliable predictive and prognostic markers. The routinely used immunochemical markers CgA, synaptophysin and CD56 have limitations of being single analytes and their presence in non-tumour tissue. Furthermore, they represent only a few of the molecules that characterise the tumour phenotype and do not include significant indicators of EMT. The ENETS group have recognised the unmet need for markers of predictive and prognostic value for tissue and blood samples [25]. Thus, we investigated a range of EMT markers to more comprehensively define NEN subtypes.

Our previous study that utilised qRT-PCR to profile a Grade 1 pancreatic neuroendocrine tumour showed substantial differences in the expression of 15 markers associated with tumour proliferation, metabolic activity, invasive potential and metastasis when tumour and matched non-tumour tissue were compared [26]. In the current study, we expanded our previous work to include thirteen patients across Grades 1, 2 and 3 and correlated the qRT-PCR results of 27 markers, including EMT-associated markers with the corresponding protein expression using IHC. The major advantage of qRT-PCR is that it is quantitative and, in contrast to IHC, which uses a small proportion of the tissue, qRT-PCR utilises a larger proportion of the sample, providing an overview of marker levels in the sample. An analysis of a larger piece of tissue would be more representative of the whole tumour tissue.

Unlike many reported studies, our study included matched controls for each patient for comparison with tumour tissue. Non-tumour marker status is not usually reported in IHC. However, measurement of the basal level of markers provides insights into the expression of markers in the tumour setting. Furthermore, markers used for cancer diagnosis can be elevated due to non-cancer health issues or associated treatments. For example, CgA is a commonly used marker to confirm a neuroendocrine origin of the tumour. However, elevated CgA serum levels have been observed due to the use of proton pump inhibitors for treatment of other disorders [27]. Hence, inclusion of matched non-tumour tissue may be warranted for routine diagnosis. It should be noted that many markers, for example, Ki67 and PCNA, indicators of cell proliferation, E-cadherin, a cell adhesion molecule and DAXX, which has many functions, are present in normal cells. Thus, it is the changes detected in marker expression levels between tumour and matched non-tumour in cancer progression that are relevant.

The main findings of our study were that mean mRNA levels of many EMT markers were consistently higher in tumour tissue relative to those in non-tumour tissue in all three grades, as well as the existing diagnostic markers CD56, CgA and Ki-67. No markers were consistently lower in tumour tissue of G3 samples relative to those in non-tumour tissue. The mRNA expression level differences between tumour and non-tumour tissues increased from G1 to G3. Seventeen markers in G3 tissue, including the EMT-associated markers CD14, CD24, CD44, CD56, CK6, CK7, CK13, CK20, NSE, CDX2, CgA, DAXX, PCNA, laminin, Ki-67 and the angiogenesis markers CD31 and CD45, were more highly expressed in tumour tissue relative to those in non-tumour tissue, while eleven markers in Grade 2 and nine markers in Grade 1 were differentially expressed in tumour relative to those in non-tumour tissue. This indicates that as the patient grade increased, the tumour tissue became more different in marker expression relative to the non-tumour tissue. This result suggests that there are multiple EMT-associated markers whose high expression relative to that in non-tumour tissue may indicate tumour progression.

Our study showed a general consistency of mRNA levels within grades for each marker, suggesting that there are similarities between patients with same-grade tumours. The current tumour grading system is based on Ki-67% and mitotic index [13]. In G2 and G3, the Ki-67 range is quite large, being 3–20% for G2 and greater than 20% for G3 and could include subgroups within each grade. NENs have the potential to be further characterised based on the markers we have used here that may enable stratification with grades.

The tissues within each grade that did not show the marker trend of their grade indicate the potential for stratification of samples within a grade that could inform diagnosis or more personalised treatment options. For example, within Grade 2, relative to that in the non-tumour tissue, the expression of markers CD24, CDX2, CD45 and CK6 was higher in Patient 1, and CD44 was higher in Patient 1 and Patient 3, compared to the other samples in the same group. Since these markers are high in G3, this may indicate that Patient 1 and Patient 3 tissues were more tumourigenic than the other G2 tissues. This could be confirmed by marker analysis of patient tissue at later stages and correlation with clinical information like metastasis to secondary sites and overall survival.

As immunohistochemistry is the accepted technique for tissue analysis, we compared the mRNA data with IHC for the same markers in tumour tissue relative to non-tumour tissue. This showed a correlation between mRNA and protein across all three grades for CgA, CD56 and Ki-67. Of the twelve markers for which the mRNA and protein levels were compared, seven in G1, eight in G2 and eight in G3 correlated, respectively, providing proof that qRT-PCR is a meaningful quantitative diagnostic tool. mRNA levels of four markers, synaptophysin (G2), CD44 (G2), DAXX (G1, G2) and CK7 (G3) did not correlate with the protein expression. These data are consistent with our previous study comparing mRNA and protein markers in a G1 pancreatic NET sample which showed that CD44 and DAXX mRNA and protein levels in G1 did not correlate [24]. Poor correlation with some CD antigens like CD24 and CD44 has also been previously reported in prostrate cell types [28]. There are several reasons for differences between mRNA and protein analysis. The biological process of transcription and translation is complex [29]. Several factors including regulatory proteins, siRNAs, translational efficiency, and protein turnover dictated by protein half-life, are involved in the process, and contribute towards mRNA–protein correlation [29,30]. It has also been reported that in a steady-state scenario, protein and mRNA levels correlate, but if cells are in a transition state, there may be differences between the mRNA and protein [31]. This could explain the number of non-correlating markers seen with the G1 and G2 samples.

mRNA levels of some markers within grades did not show significance and for these, correlation with the protein could not be determined. A larger cohort of samples would aid in achieving statistical significance.

The patterns of expression of mRNA within each grade may provide insights into the metastatic status of tumours. Based on the pathology report, the tumour sample of patient G2.1 was well-differentiated and non-metastatic (Table 1). The marker profile for this patient was different from other G2 patients, as the expression of EMT-associated differentiation markers, including CD14, CDX2, CK6, CK13 and CK20, were high, and the multifunctional EMT marker vimentin was low. The remainder of the G2 cohort tumours were metastatic and had lower levels of differentiation markers and higher vimentin expression. Thus, while the Ki-67 index of this sample placed it in G2, the morphology and vimentin levels indicated it had more features of a G1 tumour. This suggests a correlation between vimentin levels and metastatic potential of the tumour.

The EMT markers that we analysed showed variations between grades that were generally consistent with the known roles of these molecules in the EMT process underlying cancer progression. The E-cadherin-catenin complex has a dual function in cell/cell adhesion and in transcription. The loss of the E-cadherin-catenin complex from cell junctions is a major hallmark of metastatic progression and EMT [32,33]. Neuroendocrine tumours have been reported to retain β-catenin expression and E-cadherin expression in the majority of cases, while the subcellular localisation of the E-cadherin/β-catenin complex was altered and associated with lymph node metastasis [34]. The increased expression of E-cadherin in G3 tissue compared with that in G1 and G2 tissue in our study can be explained by translocation to a cytoplasmic location. A cytoplasmic location of E-cadherin has been linked to shorter survival time in cancer patients with pulmonary NETs [35], and E-cadherin is highly expressed in inflammatory breast cancer [36]. During the EMT, β-catenin undergoes translocation to the nucleus to act as a transcription factor where it mediates Wnt/β-catenin signalling in tumours, including those with neuroendocrine differentiation [37]. The higher levels of β-catenin found in our study may be a consequence of its role in the nucleus as a transcription factor where it contributes to cell proliferation, stem cell renewal and metastasis.

Vimentin expression is linked to migration and tumour cells metastasis [38]. Vimentin has been extensively investigated in breast cancer where its expression is a hallmark of EMT [39]. Less is known about vimentin in NENs. In a study of patients with Grade 1 and 2 NENs, approximately 25% tumour tissues expressed vimentin [40]. Our data showed that three out of the four G3 tumours had lower vimentin relative to that in non-tumour than the G2 tissue did. This may indicate that the G2 tumours are more actively metastatic and undergoing EMT while in G3 tissues, the metastases to secondary sites have already been established which may involve a MET. An increased epithelial phenotype has been observed in the MET prostate cancer model showing strong vimentin expression in the parental cell line, DU145, when compared to the metastatic variant DU145-LN4 [41].

CD31 is an endothelial marker present in both normal and patient blood [42,43]. Endothelial cells are sensitive to chemotherapy, and the low levels of CD31 may be due to the effects of treatment in reducing the number of vascular endothelial cells at the G3 stage of the disease [44]. Likewise with CD45, a leucocyte marker, the lower levels found in G2 and G3 may be a consequence of the chemotherapy which reduces white blood cell counts [45]. p53 mutations are associated with tumour metastasis, but tissue from gastrointestinal NEN patients p53 immunoreactivity was only shown in less than 40% of the cases and was not found to be a prognostic marker [46].

Tumourigenesis is accompanied by cellular changes in different categories of EMT markers. Thus, we grouped the markers according to their categories, including proliferation, cell adhesion, altered cytoskeleton, cell migration and metastasis, cellular de-differentiation and tumour suppression to determine how they varied across Grades 2 and 3 relative to a G1 tumour as a baseline. It excluded the relative mRNA changes between tumour and non-tumour tissue. The markers that distinguished G3 tumours from the lower grades were higher levels of synaptophysin, CD56, Ki-67, CD24, CD44, β-catenin and E-cadherin. Markers that were lower in G3 compared to G1 and G2 included CgA, CD31, CD45, vimentin, laminin, CD14 and CDX2. The markers that distinguish G2 tumours from G1 or G3 included higher expression of CD31, vimentin, CD44, laminin and p53 and lower expression of CD56, CD24, PCAD and PDX1.

The nine most relevant markers from each category can be visualised in the form of a molecular signature that indicates the profile differences of between the grades. The nine markers include CgA, NSE (NE origin), Ki-67, DAXX (proliferation), CD24, CD44 (stem cell), vimentin (EMT), laminin (cell adhesion) and PDX1 (differentiation). Two of these are neuroendocrine-specific markers, while seven are EMT-related. This collection constitutes a biomarker panel for neuroendocrine tumours indicating tumour progression from Grade 1 to Grade 3. It demonstrates the link between tumour progression and validates the link between expression of EMT markers and tumour progression. The utility of biomarker panels has previously been shown in breast cancer to predict response to taxane-based neoadjuvant chemotherapy [47] and in the application of mRNA signatures to identify novel biomarkers that predict the prognosis of patients with non-small-cell lung cancers [48]. Prognostic markers associated with a good outcome, defined as lack of progression to G3, include high expression levels of CgA and PDX1 and low expression levels of Ki67, NSE, DAXX, CD24, CD44, laminin and PDX1.

The main limitation of the study was the limited number of samples. Nevertheless, with a relatively small sample size, consistent patterns in the expression of biomarkers were obtained that enabled characterisation of tumour phenotypes between grades.

## 4. Materials and Methods

### 4.1. Sample Collection and Classification

Pancreatic tumour specimens with corresponding matched adjacent non-tumour tissue from thirteen patients with pancreatic neoplasms including four G1, five G2 and four G3 samples were obtained from the Kolling Institute Tumour Bank and the Victorian Cancer Biobank and stored at −80 °C until use. Regions of tissue showing normal pancreatic morphology were classed as non-tumour, and regions where the tissue was disorganised, containing cells with irregular nuclei and heterogeneity of size, were classified as tumour tissue [49]. Table 3 shows the de-identified histopathology details of each patient sample including the Ki-67%, grade, tumour location and metastasis status. The grading of biobank tissues was based on IHC analysis of Ki-67. The location of the tumour varied from the head, neck, body to the tail of the pancreas. In terms of metastatic status, one G1 patient presented with lymph node invasion, four G2 patients presented with invasion in the lymph nodes and/or liver and four patients G3 presented with invasion in the lymph nodes and/or duodenum.

### 4.2. Selection of Tumour-Associated Markers

Twenty-seven markers that correlated with a range of tumour-associated characteristics that had been previously tested in a pancreatic tumour case study [24] were selected (Table 4). These markers included indicators of a neuroendocrine origin, a category of markers associated with EMT including proliferation, stem cell phenotype, cell adhesion, differentiation and tumour suppression, a multifunctional category that included EMT-associated markers and a category of vascular markers indicating angiogenesis.

### 4.3. Ethics

The experimental protocol received an ethics approval from Deakin University STEC-09-2018 ACKLAND.

### 4.4. Quantitative Real-Time Reverse Transcription PCR (qRT-PCR)

#### RNA Extraction

A 2 mm × 2 mm frozen tissue was cut and ground using a mortar and pestle in the presence of liquid nitrogen and further homogenised by passing through a 20-gauge needle and syringe multiple times. RNA was extracted from the tissue samples using an RNeasy mini kit (Qiagen) as per the manufacturer’s instruction. RNA concentrations were measured using a Thermo Scientific NanoDrop 1000. Only RNA that passed the QC of 260/280 ratio of 2 was reverse transcribed into cDNA.

### 4.5. Reverse Transcription

RNA was converted into cDNA using an Applied Biosystems High-Capacity cDNA Reverse Transcription Kit as per the manufacturer’s instructions. A working solution of 5 ng/uL was prepared by diluting the stock using nuclease-free water.

### 4.6. Real-Time PCR

Ten milliliters of PowerUp SYBR Green Master Mix (Applied Biosystems), 5 ng of cDNA template, 250 nM of each primer was made up to 20 mL in a PCR reaction/well using nuclease-free water. A template (20 ng) was used for markers which were expressed in very low levels. GAPDH and b-actin were used as the endogenous control. Each run also included negative controls of a master mix without cDNA to rule out contamination. The PCR cycle was 50 °C—2 min, 95 °C—10 min and 45 cycles of 95 °C—15 s, 60 °C—1 min and a final extension for 95 °C—15 s. The plate was loaded onto a Bio-Rad CFX96 qPCR machine, and the marker levels were analysed using the relative quantification method (ΔΔCt). All markers were tested for 3 individual qPCR runs, and triplicate experiments were run for each sample. The fold change was calculated using the 2^−ΔΔCt^ formula. mRNA transcript levels of each marker were measured in tumour tissue relative to matched non-tumour tissue. The relative expression of markers was first expressed as fold change calculated using b-actin as the endogenous control. Tumour marker mRNA levels were then expressed relative to the control non-tumour tissue which was given an mRNA expression value of one.

### 4.7. Immunohistochemistry (IHC)

Tissue Sectioning: Tissue samples of size 3 mm × 3 mm were cut and frozen in liquid nitrogen in OCT. Sections of 5–8 mm thickness were sectioned and collected on a charged slide using a Thermo Scientific HM525NX cryostat. The slides were air-dried and stored at −80 °C until use.

### 4.8. Immunostaining

Frozen sections were rinsed three times with 1× PBS for 10 min then fixed in 4% paraformaldehyde (PFA). The slides were washed three times with 1X PBS then incubated in 0.3% hydrogen peroxide for 10 min followed by 10 min incubation with 0.1% Triton × with three 1× PBS washes between the two incubations. The sections were blocked in 2% BSA for 45 min and then incubated with primary antibodies at 4 °C overnight. The following day, the slides were washed using 1× PBS and incubated with horseradish peroxidase or alkaline phosphatase enzyme tagged secondary antibody for 30 min. The corresponding substrate, ImmPACT DAB or alkaline phosphatase (Vector Laboratories) was then added, followed by counterstaining with methyl green or haematoxylin and mounting using a DPX solution (Sigma Aldrich).

### 4.9. Imaging

Images were taken at 40×, 100× and 200× magnifications using an Olympus BX43 light microscope fitted with an Olympus DP22 camera. Images were collected using DP software and analysed by comparing the intensity of the label in the tumour sample to that of the matched non-tumour tissue.

### 4.10. Statistics

#### 4.10.1. *t*-Test

A two-tailed *t*-test with unequal variance was used to find the probability value (*p*-value) between each normal and tumour sample and its positive and negative standard deviation values. *p*-values less than 0.05 were considered statistically significant. The average of the relative fold change and standard deviation of the samples from the same grade were used to calculate the marker expression profile for each grade.

#### 4.10.2. One-Way ANOVA

Marker expression was studied across tumour grades using G1 as the reference sample. One-way ANOVA with post-hoc Tukey’s test was used, and markers with a *p*-value < 0.05 were considered significant. “a” and “b” represent markers expressed significantly differently compared to G1 and G2.

## 5. Conclusions

Tissue from patients with Grade 1, 2 and 3 tumours had distinct profiles seen in the expression of a range of EMT markers. Most EMT markers had consistently higher mRNA levels in tumour tissue relative to those in non-tumour tissue in all three grades, with expression levels increasing with higher grade. The differences in tumour markers between grades confirmed the link between EMT and tumourigenesis and provided insights into the molecular changes associated with cancer progression. The marker profiles can be visualised as a molecular signature of nine markers, CgA, NSE (NE origin), Ki-67, DAXX (proliferation), CD24, CD44 (stem cell), vimentin (multifunctional), laminin (cell adhesion) and PDX1 (differentiation), that have potential for use in tumour stratification and for identification of clinically relevant subtypes. The value of such a molecular signature needs to be tested in a larger cohort of patients.

## Figures and Tables

**Table 1:** mRNA levels of 27 markers in tumour tissue relative to matched non-tumour using β-actin as an endogenous control.

Grade 1	CgA	Syn	NSE	CD56	Ki67	PCNA	DAXX	CD24	CD44	CD31	CD45	Vim	β−Cat	MENA	CD49	Lam	ECAD	PCAD	EpCAM	CDX2	PDX1	CD14	CK6	CK7	CK13	CK20	P53
**1**	− *	−−	−−	−− *	−	−−	−− *	− *	−− *	−	−− *	+	++ *	−	−− *	−− *	−	−− *	−	−−− *	+ *	−− *	−−− *	−−− *	−−	−−−	++ *
**2**	++ *	+	−	++ *	++ *	=	−− *	−−−	−−	−−	−−	−−	−−	+	−−	−−− *	+	−	++	−−	++ *	−−	−−	++	−	−−−	+
**3**	+++ *	−	++	++ *	+ *	−	−− *	−− *	−− *	−− *	−− *	−− *	− *	++	−−− *	−−− *	++ *	−	++	−−− *	+++ *	− *	−−− *	−−	−−−	−−−	+ *
**4**	+++ *	=	+	++ *	−−− *	=	=	− *	− *	−− *	+ *	+	++ *	−− *	− *	=	−− *	−−− *	−− *	−− *	++ *	=	−− *	−−− *	− *	++ *	++ *
**Grade 2**	**CgA**	**Syn**	**NSE**	**CD56**	**Ki67**	**PCNA**	**DAXX**	**CD24**	**CD44**	**CD31**	**CD45**	**Vim**	**β−Cat**	**MENA**	**CD49**	**Lam**	**ECAD**	**PCAD**	**EpCAM**	**CDX2**	**PDX1**	**CD14**	**CK6**	**CK7**	**CK13**	**CK20**	**P53**
**1**	+++ *	−−	++	+	=	−	+	+ *	+ *	+ *	+ *	−−	−−− *	−− *	+ *	=	−−	−−− *	++	++ *	−	++ *	++ *	−−− *	++ *	++ *	−−
**2**	++ *	− *	+++	++ *	− *	=	− *	−−− *	−− *	++ *	− *	+ *	−− *	=	− *	+ *	−− *	−− *	+	− *	−−− *	+ *	−	−−− *	+	+	− *
**3**	+ *	+	++ *	++ *	++ *	++ *	−	−− *	+ *	+ *	−−− *	++ *	− *	++ *	+ *	−	++ *	− *	++ *	− *	−−− *	− *	+	− *	+	−−	−
**4**	++ *	− *	−−	+++ *	+++ *	−	−− *	−− *	−− *	−	−− *	++ *	+	+	−− *	−	++ *	+++	−	−− *	−− *	−	− *	−−− *	− *	−−	+
**5**	++ *	−− *	−−	−	++ *	−− *	−− *	+	−− *	−−	−−− *	++ *	+	=	−− *	− *	−− *	−− *	−− *	−− *	−−	−− *	− *	+	−−− *	−−	++ *
**Grade 3**	**CgA**	**Syn**	**NSE**	**CD56**	**Ki67**	**PCNA**	**DAXX**	**CD24**	**CD44**	**CD31**	**CD45**	**Vim**	**β−Cat**	**MENA**	**CD49**	**Lam**	**ECAD**	**PCAD**	**EpCAM**	**CDX2**	**PDX1**	**CD14**	**CK6**	**CK7**	**CK13**	**CK20**	**P53**
**1**	++ *	−− *	+	++ *	++ *	+	−− *	++ *	++ *	−− *	−−− *	−− *	− *	−	−− *	+	−− *	−− *	−	−−− *	−	=	−−− *	−− *	−−−	−−− *	−
**2**	++ *	− *	++ *	++ *	++ *	++ *	−	+ *	+	−− *	− *	−− *	+	−− *	−− *	− *	++ *	++ *	+	−	+	=	− *	+	−− *	−− *	+ *
**3**	=	+	+++	++ *	+ *	+	++ *	++ *	++ *	++ *	+++ *	++ *	+	+	++ *	+++ *	−− *	++ *	+	++ *	=	++ *	+++ *	+++ *	+++ *	−	−− *
**4**	+++ *	−	++ *	++ *	++ *	++ *	++ *	−− *	++ *	+ *	++ *	−	−	−− *	−−	++ *	−−− *	−	−	+++ *	−− *	++ *	++ *	−− *	++ *	+++ *	++ *

Tissue from 13 patients was grouped into Grade 1, Grade 2 and Grade 3. + and − indicate whether marker mRNA transcript levels were higher or lower in the tumour tissue compared to that in the matched non-tumour tissue. * represents significant changes (*p* < 0.05). −:1–2 fold lower compared to non-tumour; −−: 2–10 fold lower compared to non-tumour; −−−: >10 fold lower compared to non-tumour; +: 1–2 fold higher relative to non-tumour; ++ 2–10 fold higher relative to non-tumour; +++ >10 fold higher relative to non-tumour; = similar in tumour and non-tumour. Vim: vimentin and Lam: laminin.

**Table 2:** Table comparing mRNA and protein levels for selected markers.

Marker	mRNA			Protein		
	G1	G2	G3	G1	G2	G3
**CgA**	+++ *	+++ *	++ *	++	+	++
**Syn**	−	− *	−	+	+	+
**CD56**	++ *	++ *	++ *	++	+	+
**Ki67**	+ *	++ *	+++ *	+	+	++
**DAXX**	−− *	− *	++ *	=	=	+
**CD44**	−−	− *	++ *	++	=	+
**CD31**	−−	+	+ *	+	++	++
**Vimentin**	−	++ *	−	=	++	=
**Laminin**	−− *	−	++ *	−−	−−−	+
**PDX1**	+++ *	−− *	−	++	−	=
**CK7**	−	−− *	+++ *	−−−	−−	−−
**P53**	++ *	+	+	++	=	+

For mRNA levels: + and − indicate whether marker mRNA transcript levels were higher or lower in the tumour tissue compared to that in the matched non-tumour tissue. * represents significant changes (*p* < 0.05). −: 1–2 fold change lower compared to normal; −−: 2–10 fold change lower compared to normal; −−−: >10 fold change lower compared to normal; +: 1–2 fold change higher relative to normal; ++: 2–10 fold change higher relative to normal; +++: >10 fold change higher relative to normal; =: similar in tumour and non-tumour. For protein levels: + and − indicate whether protein expression was higher or lower in the tumour tissue compared to that in the matched non-tumour tissue. If present, +: <25% of the field, ++: 25–50% of the field and +++: >50% of the field stained for the marker. −: <25%, −−: 25–50%, −−−: >50% less expression in the tumour tissue relative to that in non-tumour.

**Table 3:** Samples and their pathological report detailing Ki67%, grade, tumour location and metastatic status.

Sample Id	Ki67 (%)	Mitotic Index (per 10 hpf)	Grade	Tumour Location	Metastasis
G1.1	0.5	<1	G1 (NET)	Pancreatic neck (Whipple’s)	No.
G1.2	1	Infrequent 1	G1 (NET)	Distal Pancreas	Yes, 1/4 peri pancreatic lymph nodes
G1.3	1	Upto 1 (<2)	G1 (NET) well differentiated	Pancreas (Whipple’s)	No. History of MEN type 1
G1.4	<2%	Not seen in 50hpf	G1 (PEN) well differentiated	Distal Pancreas	No
G2.1	4–5	1	G2 (NET) well differentiated	Distal Pancreas	No.
G2.2	10	2	G2 (NET)	Distal pancreas	Yes, 3/18 lymph nodes, secondary liver
G2.3	10	2	G2 (NET) moderately differentiated	Pancreas (Whipple’s)	Yes, 2/11 nodes
G2.4	10–15	7/50 hpf	G2 (NET)	Distal Pancreas	Yes, liver
G2.5	15–20	4	G2 (NET)	Pancreas head	Yes, mesenteric lymph node
G3.1	15–30%	1	G3 (NET)	Pancreas head and uncinate	Invasion into duodenum and lymph nodes
G3.2	35	Generally, 10 One area 30	G3 (NET)	Pancreatic head and neck	Yes, 6/24 lymph nodes
G3.3	40	19	G3 (NET)	Pancreas (Whipple’s)	Yes, 4/11 lymph nodes
G3.4	50	31	G3 (NET)	Pancreatic tail	Yes, 5/13 nodes involved

**Table 4:** List of marker categories, classes and names.

Category	Marker Class	Marker
Neuroendocrine	Neuroendocrine	Chromogranin A (CgA), synaptophysin, neuron specific enolase (NSE), CD56
Epithelial to Mesenchymal Transition (EMT)-associated	Proliferation	Ki-67, PCNA, DAXX
	Stem Cell	CD24, CD44, CD49 (Integrin alpha-6)
	Cell Adhesion	Laminin, E-cadherin (ECAD), P-cadherin (PCAD), EpCAM, CD56, CD49
	Differentiation	CDX2, PDX1, CD14, CK6, CK7, CK13, CK20
	Tumour suppression	P53
Multifunctional including EMT		Vimentin, β-catenin, MENA
Vascular/haematopoietic	Angiogenesis	CD31, CD45

## Data Availability

Raw data are available from the corresponding author M Leigh Ackland (leigha@deakin.edu.au).
